# An innovative way to modulate the photoluminescence of carbonized polymer dots

**DOI:** 10.1038/s41377-022-00772-1

**Published:** 2022-03-30

**Authors:** Pengfei Li, Zaicheng Sun

**Affiliations:** Center of Excellence for Environmental Safety and Biological Effects, Beijing Key Laboratory for Green Catalysis and Separation, Faculty of Environment and Life, Beijing University of Technology, Beijing, 100124 P. R. China

**Keywords:** Polymers, Fluorescence spectroscopy

## Abstract

Cross-linking enhances the photoluminescence quantum yield of carbonized polymer dots, in which confined-domain promotes the energy level overlap, redshifts emission wavelength, and facilitates phosphorescence generation.

Carbon dots (CDs) were first observed during the preparation of single-walled carbon nanotubes in 2004. And then in 2007, CDs were defined in detail as zero-dimension carbon nanomaterials with diameters of 2–10 nm. It possesses wide and fantastic potential applications in optoelectronic devices, biomedicine, photocatalysis, etc. as the excellent optical properties, good biocompatibility, simple preparation. Thus, it also becomes hot material in a lot of fields. Depending on the composition and structure of the CDs, they can be divided into graphene quantum dots (GQD), graphitic carbon nitride quantum dots (CNQD), carbon quantum dots (CQDs), carbon nanodots (CNDs), and carbonized polymer dots (CPDs).

CDs have been rapidly developed and made great achievements in the last decade, various researchers put enormous enthusiasm and energy to regulate their optical properties. Up till the present, CDs can achieve full spectral emission and the maximum emission wavelength can reach far IR. However, it is still far from practical application. The major cause is the controversial photoluminescence (PL) mechanism. As is well known “properties depend on the structure”, without doubt, the optical properties of CDs also depend on the structure. Therefore, the intricate and ambiguous structures naturally become the greatest hindrances in the process of researching the CDs PL mechanism.

Of course, the PL mechanism has been studied since CDs were discovered, the carbon core state, surface state, and molecule state as the mainstream views are widely recognized by researchers^1,2^. The core state fluorescence caused by π–π* electron transitions, the size of isolated sp^2^ subdomain determines PL property^3,4^. This PL mechanism is extensively applicable for CDs that possess large pure sp^2^ carbon crystalline structure sizes. The surface state is considered that surface functional groups regulate the electronic structures and energy levels of CDs to control the bandgap of CDs, to lead the PL^5,6^. This type of CDs usually has the conjugated carbon backbone and the surface contains abundant functional groups. The molecule state refers to molecular fluorophores carried by CDs dominating the PL property, it is often formed in the early stage of the synthesis process by bottom-up routes. Moreover, the type of CDs through the preparation method are CQDs, CNDs, and CPDs^2,7^. However, the aforesaid cannot explain the unconventional PL behaviors exhibited by the sub-luminophores in non-conjugated polymer dots, the unconventional PL behaviors always present to CQDs. Therewith Yang et al. proposed the pioneering concept of cross-link-enhanced emission effect (CEE) to reveal the PL behaviors, which has been widely applied and exploited^8^.

Generally, some electron-rich heteroatom functions groups (such as C=O, C=N, N=O, and N–O) transition back to ground states from the excited states through the non-radiative pathways without luminescence. In the CEE PL mechanism (Fig. 1), these electron-rich heteroatom functions groups defined as sub-luminophore, it is immobilized by cross-linking polymers to restrict the vibration and rotation of sub-fluorophores, leading to the increase of radiative transition and decrease of the non-radiative transition, thereby enhancing the sub-fluorophores PL of CPDs^9,10^. Nevertheless, this only improves the quantum yield (QY) of CPDs but does not explain the change of the energy level and the conversion of the radiative process from fluorescence to phosphorescence. In a recent publication by Tao et al.^11^, the confined-domain CEE effect on luminescence properties of CPDs was investigated deeper perspective by combining characterizations and theoretical calculations, revealing the photophysical process.

**Fig. 1:**
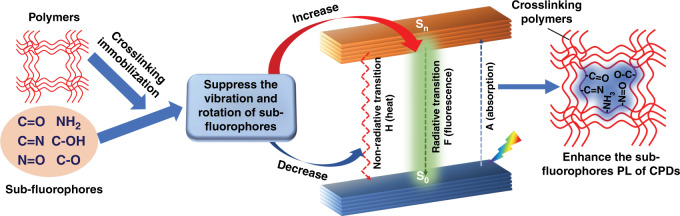
Schematic of cross-link-enhanced emission effect. Cross-linking restricts the vibration and rotation of sub-fluorophores (heteroatom-containing double bonds (C=O, C=N, N=O) and single bonds (amino based groups, C–O)), leading to the increase of radiative transition and decrease of the non-radiative transition, thereby enhancing the sub-fluorophores PL of CPDs (S_0_: Ground singlet state, S_n_: Excited singlet state)

Figure 2 shows the shame of confined-domain CEE effect on energy level and electron transition, stable and compact environment of CPDs can greatly promote the electron-cloud overlaps and couple, split intrinsic energy levels, generates sub-levels, resulting in emission wavelength shift. In addition, The coupled units also possible to narrow the energy gap between the excited singlet state and triplet excitons state (△E_S-T_), which change the pathway of radiative transitions, facilitated intersystem crossing (ISC) and triplet generation. Herein, the CPDs system model with different strengths of confined-domain CEE was constructed by modulating the content of methyl groups in copolymers. Increasing methyl groups content weakened the cross-linking degree and strength of confined-domain CEE, but they process the same PL centers (bear amide or imide groups). From the above of femtosecond transient absorption (TA) spectroscopy, the great degree cross-linking CPDs appeared more energy-level structures at excited state, indicating that confined-domain CEE stimulates the production of abundant sub-levels, increase the number of transition channels through space interaction, which is a benefit to PL. The theoretical calculations studied the spin-orbit coupling (SOC) coefficients which can quantitatively estimate the probability of ISC and triplet emission. The results suggested that the generation ability of the triplet excitons and the extent of phosphorescence emission gradually decreased with the strength weakened of confined-domain CEE, weakened room-temperature phosphorescence (RTP) performance of CPDs. This is possible because the confined-domain CEE upshifts the triplet state’s energy level, narrowing the △E_S-T_, and making the ISC easier. Furthermore, this also can extend the phosphorescence lifetime^12^.

**Fig. 2:**
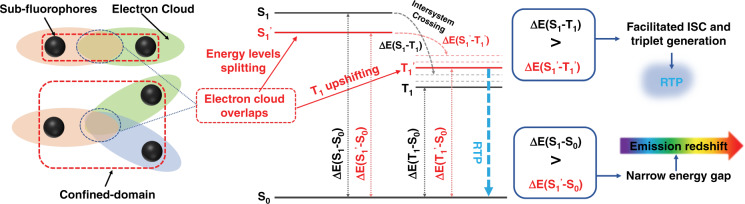
Schematic showing the confined-domain cross-link-enhanced emission effect. The overlap and couple of electron clouds caused by confined-domain split intrinsic energy levels to sub-levels and upshift the triplet states energy level. This results in PL emission redshift and RTP generation. (S_0_: Ground singlet state, S_1_: The original excited singlet state, T_1_: The original excited triplet states, S_1_: The formed excited singlet state from energy levels splitting, T_1_′: The formed excited triplet state from T_1_ upshifting, ΔE(S_1_–S_0_): The original energy gap between S_0_ and S_1_, ΔE(S_1_′–S_0_): Energy gap after confined-domain cross-link between S_0_ and S_1_′, ΔE(S_1_–T_1_): The original energy gap between S_1_ and T_1_, ΔE(S_1_′–T_1_′): Energy gap after confined-domain cross-link between S_1_′ and T_1_′)

The confined-domain CEE involved multiple photophysical processes. Based on the concept of confined-domain CEE, the CPDs emission of both fluorescence and phosphorescence can be modulated and targeted synthesis. More and more diverse features and extensive functions can be developed, for example, tuning the RTP lifetimes of CPDs by confined-domain CEE can develop the potentially smart materials apply to multi-level anti-counterfeiting technology^13,14^. However, Variability in the properties of CPDs arising from methyl groups is relatively limited as methyl group is simple in structure. A variety of other substituents, such as aromatic rings and halogens, can be introduced into the structures of precursors for developing novel CPDs^15^.
